# Mechanical-Thermal Noise in Drive-Mode of a Silicon Micro-Gyroscope

**DOI:** 10.3390/s90503357

**Published:** 2009-05-07

**Authors:** Bo Yang, Shourong Wang, Hongsheng Li, Bailing Zhou

**Affiliations:** College of Instrument Science & Engineering, Southeast University, Nanjing 210096, China; E-Mails: srwang@seu.edu.cn; hsli@seu.edu.cn; zhoubailing@seu.edu.cn

**Keywords:** Silicon Micro-Gyroscope (SMG), drive-mode, thermal noise, stochastic averaging

## Abstract

A new closed-loop drive scheme which decouples the phase and the gain of the closed-loop driving system was designed in a Silicon Micro-Gyroscope (SMG). We deduce the system model of closed-loop driving and use stochastic averaging to obtain an approximate “slow” system that clarifies the effect of thermal noise. The effects of mechanical-thermal noise on the driving performance of the SMG, including the noise spectral density of the driving amplitude and frequency, are derived. By calculating and comparing the noise amplitude due to thermal noise both in the opened-loop driving and in the closed-loop driving, we find that the closed-loop driving does not reduce the RMS noise amplitude. We observe that the RMS noise frequency can be reduced by increasing the quality factor and the drive amplitude in the closed-loop driving system. The experiment and simulation validate the feasibility of closed-loop driving and confirm the validity of the averaged equation and its stablility criterion. The experiment and simulation results indicate the electrical noise of closed-loop driving circuitry is bigger than the mechanical-thermal noise and as the driving mass decreases, the mechanical-thermal noise may get bigger than the electrical noise of the closed-loop driving circuitry.

## 1. Introduction

The Silicon Micro-Gyroscope (SMG) is an important MEMS inertia sensor with a broad application in the national economy and defense fields [1-7]. These SMGs usually measure the proof-mass displacement by capacitive methods, but under normal atmospheric pressure, the minute moving proof-masses are especially susceptible to mechanical noise resulting from molecular agitation. Although accuracy is usually limited by electrical noise and systematic errors, mechanical thermal noise provides a theoretical lower limit for random errors [8-11]. Thus, a proper accounting of thermal noise is essential for the development of higher accuracy tactical and inertial grade gyroscopes.

The effects of mechanical-thermal noise on the sense-mode have been presented in the literature [8-11], but discussions of the effects of mechanical-thermal noise on drive-mode can hardly be found in the current literature. In this paper the effects of mechanical thermal noise on the driving performance of the SMG are mainly derived. Only the influence of the mechanical thermal noise is considered, while the electrical noise, sampling and quantization error, and distortion due to filtering are not considered. Meanwhile, we assume all the other processes run in an ideal manner. In this paper, a stochastic averaging approach is used to take account of the effects of closed-loop drive. The effect of mechanical thermal noise on drive-mode is discussed, then stochastic averaging is used to develop a model for the “slow” dynamics which represent the driving amplitude and frequency of the SMG. Both the steady-state and transient response of the model are obtained by stochastic averaging. The spectral density of the random error due to thermal noise on drive-mode is also derived.

## 2. Working Principle

**Figure 1.**
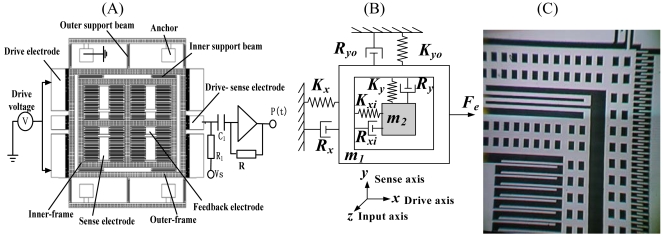
(A) The frame of the SMG. (B) The simple model of SMG. (C) The picture of the processed SMG.

As shown schematically in Figure 1(A), the micro-gyroscope consists of two silicon frames (outer-frame and inner-frame); the outer-frame is anchored on a glass substrate by six outer support beams and is connected with the inner-frame through four inner support beams. The outer-frame and the fixed interdigitated drive electrode on the glass substrate form the drive capacitors. The alternating drive force of the out-frame along the x-axis is generated through applying alternating current (AC) voltage with direct current (DC) bias voltage to the fixed drive electrode. Since the stiffness of the inner support beam along the x-axis (K_xi_≫ K_x_) is very large, the outer-frame and the inner-frame are driven together to vibrate along the x-axis by the alternating drive force, which causes the alternating capacitance between the outer-frame and fixed drive-sense electrode. We can capture the drive displacement by detecting the alternating capacitance. When the rotation rate along the z-axis is input, according to the Coriolis effect, the Coriolis force along the y-axis will be loaded on both the outer-frame and the inner-frame. Because the stiffness of the outer support beam along the y-axis (K_yo_≫K_y_) is very large, only the inner-frame is driven to vibrate along the y-axis by the Coriolis force, which induces the alternating capacitance between the inner-frame and fixed sense electrode. We can obtain the rotation rate along the z-axis by detecting the alternating capacitance.

The simplified motion equations of SMG are described by:

(1) $$m_{x}\ddot{x}+R_{x}\dot{x}+K_{x}x=F_{e}+n$$

(2) $$m_{y}\ddot{y}+R_{y}\dot{y}+K_{y}y=-2m_{y}\Omega \dot{x}$$

where x and y are separately the drive axis displacements and sense axis displacements in meters, Ω the rotation rate along the z-axis in radians/second, m_x_ (m_x_=m_1_+m_2_) and m_y_ (m_y_=m_2_) the drive proof mass and the sense proof mass in kilograms, R_x_ and R_y_ the damping in Newtons/meter/second, K_x_ and K_y_ the stiffness in Newtons/meter, and −2*m _x_*Ω*ẋ* the denote of the Coriolis force. F_e_ (F_e_=F_d_sinω_d_t) is the electrostatic force used to maintain the drive-mode vibration at a specified amplitude in terms of displacement, and at a resonant frequency of the drive-mode. Mechanical thermal noise on the drive axis is represented by the random force n(t), in units of force.

Ignoring the influence of the random force n(t), the drive axis displacements and sense axis displacements in the steady state are described by:

(3) $$x\approx A_{x}\sin(\omega_{d}t+\varphi )$$

(4) $$y\approx A_{y}\sin(\omega_{d}t+\varphi +\gamma )$$

where $A_{x}=\frac{F_{d}}{m_{x}\sqrt{{\left(\omega_{nx}^{2}-{\omega_{d}}^{2}\right)}^{2}+\frac{\omega_{nx}^{2}{\omega_{d}}^{2}}{Q_{x}^{2}}}}$; $\varphi =-tg^{-1}(\frac{\omega_{nx}\omega_{d}}{Q_{x}(\omega_{nx}^{2}-{\omega_{d}}^{2})})$; $A_{y}\approx \frac{-2\Omega \omega_{d}A_{x}}{\sqrt{{\left(\omega_{n\text{y}}^{2}-{\omega_{d}}^{2}\right)}^{2}+\frac{\omega_{ny}^{2}{\omega_{d}}^{2}}{Q_{y}^{2}}}}$; $\gamma =tg^{-1}(\frac{Q_{y}(\omega_{ny}^{2}-{\omega_{d}}^{2})}{\omega_{d}\omega_{ny}})$; ω_nx_ =(K_x_/m_x_)^(1/2)^; ω_ny_ =(K_y_/m_y_)^(1/2)^; Q_x_=m_x_ω_nx_/R_x_, Q_y_=m_y_ω_ny_/R_y_.

When ω_d_=ω_nx_=ω_ny_, the maximum drive axis displacements and sense axis displacements are described by:

(5) $$x(t)=\frac{F_{d}Q_{x}}{K_{x}}\sin(\omega_{d}t-\frac{\pi }{2})$$

(6) $$y(t)=\frac{-2F_{d}Q_{x}Q_{y}\Omega }{K_{x}\omega_{d}}\sin(\omega_{d}t-\frac{\pi }{2})$$

## 3. Mechanical Thermal Noise

Consider the damped harmonic oscillator:

(7) $$m_{x}\ddot{x}+R_{x}\dot{x}+K_{x}x=n$$

The presence of damping in the system suggests that any oscillation would continue to decrease in amplitude forever. Inclusion of the fluctuating force n(t) prevents the system temperature from dropping below that of the system's surroundings. The damper provides a path for energy to leave the mass-spring system. This is the essence of the Fluctuation-Dissipation Theorem. According to Equipartition, if any collection of energy storage mode is in thermal equilibrium, then each mode will have an average energy equal to (1/2)k_B_T where k_B_ is Boltzmann's constant(1.38×10^-23^J/K) and T is the absolute temperature in degrees Kelvin. A mode of energy storage is one in which the energy is proportional to the square of some coordinate; e.g., kinetic and spring potential.

When this system is in thermal equilibrium, the probability distribution of x and *ẋ* is given by equation (8):[12]

(8) $$p(x,\dot{x})=\text{constant}\times e^{-\frac{E(x,\dot{x})}{k_{B}T}}$$

For the oscillator, the energy is the sum of the kinetic and spring potential energy:

(9) $$E(x,\dot{x})=\frac{1}{2}m_{x}{\dot{x}}^{2}+\frac{1}{2}K_{x}x^{2}$$

From here, the equipartition theorem can be derived, namely that the mean energy in any energy storage mode is equal to(1/2)k_B_T. Thus:

(10) $$<\frac{1}{2}m_{x}{\dot{x}}^{2}>=<\frac{1}{2}K_{x}x^{2}>=\frac{1}{2}k_{B}T$$

where <·>denotes an ensemble average. The form of the distribution *p*(*x, ẋ*) indicates that *x* and *ẋ* are independent, Gaussian, and have zero mean. Since this holds for all values of m_x_ and K_x_, the thermal noise n(t) must be a white Gaussian noise with two sided spectral density [10]:

(11) $$S_{n}(\omega )=2k_{B}TR_{x}{\text{N}}^{2}/\text{Hz}-\infty <\omega <+\infty$$

The spectral density of drive displacements due to thermal noise is:

(12) $$S_{x_{n}}(\omega )=\frac{1}{{m_{x}}^{2}\left({\left(\omega_{nx}^{2}-\omega^{2}\right)}^{2}+\frac{\omega_{nx}^{2}\omega^{2}}{Q_{x}^{2}}\right)}S_{n}(\omega )$$

So the noise power spectrum is:

(13) $$<x_{n}^{2}>=\frac{1}{2\pi }\int_{-\infty }^{+\infty }S_{x_{n}}(\omega )d\omega =\frac{k_{B}T}{K_{x}}$$

The RMS noise displacements due to thermal noise is:

(14) $$x_{n}=\sqrt{<x_{n}^{2}>}=\sqrt{\frac{k_{B}T}{K_{x}}}=\sqrt{\frac{k_{B}T}{m_{x}{\omega^{2}}_{nx}}}$$

According to Equation (5) and Equation (14) the signal-to-noise ratio of drive-mode is:

(15) $${\left(S/N\right)}_{x}=x/x_{n}=\frac{F_{d}Q_{x}}{\sqrt{K_{x}k_{B}T}}$$

The above equation indicates that we can improve the signal-to-noise ratio by increasing the quality Q_x_ and driving force amplitude F_d_, or by reducing the stiffness and temperature.

## 4. Closed-loop Driving and System Model

As is known in the art of Coriolis force sensors, in order to achieve an acceptable response from the sensor, the proof mass vibration of the drive-mode should have a frequency at, or close to, the resonant frequency of the proof mass. At the same time, in order to improve the entire performance of the SMG, a high stability of the driving frequency and the amplitude of the drive-mode are needed. To satisfy those demands, the closed-loop driving of the drive-mode must be achieved. To this end, the drive signal has a frequency equal to the resonant frequency of the proof mass. However, parasitic capacitances between the drive electrode and the drive-sense electrode can cause significant errors. That is, when the drive signal capacitively couples into the drive-sense electrode, the accuracy of amplitude control by the feedback circuit is degraded and the harmonic frequency of the closed-loop system departs from the resonant frequency of the proof mass, resulting in less than optimum sensor performance, so we must eliminate the capacitive coupling. Various techniques are generally utilized in an effort to reduce capacitive coupling. In this paper, such a technique is utilized as follows: the drive electrode is arranged on the left, the drive-sense electrode is arranged on the right and the anchor of the SMG is connected with the ground or the virtual ground, which is shown in Figure 1(A). By separating the drive-sense interface (drive-sense electrode) from the interference source (drive voltage), we can reduce these capacitive couplings. Besides, the SMG studied in this paper is executed in vacuum encapsulation, with a working pressure under 10^-1^ Torr and the quality factor of the drive-mode above 2,500, which can also reduce these capacitive coupling by decreasing the drive voltage. The modulation-demodulation method through applying high-frequency carrier to the proof mass can also reduce these capacitive couplings.

**Figure 2.**
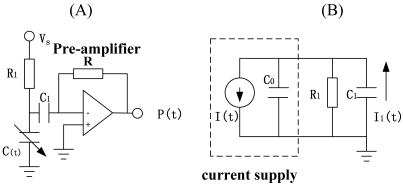
(A) The interface circuit of drive-sense signal. (B) The equivalent circuit.

First, we need to extract the resonance signal of the drive-mode. The simplified interface circuitry is shown in Figure 1. Figure 2 show the details of the interface circuit and the equivalent circuit. Here in Figure 2, C (t) is the alternating drive-sense capacitance, C(t)=C_0_+ΔC, C_0_ is constant capacitance. The part of signal sense can be equivalent to a current supply I(t) and a internal resistance C_0_ [see Figure 2(B)], where:

(16) $$I(t)=\frac{dQ}{dt}=\frac{d(V_{s}C(t))}{dt}=V_{d}K_{xc}\dot{x}$$

In Equation (16), the differential of driving capacitance to displacement K_xc_ ${(\text{K}}_{\text{xc}}=\frac{\partial C}{\partial x})$ is a constant relating to the structure. The capacitance C_0_ is very minute and generally has hundreds of fF, thus the impedance of C_0_ is very large and we can ignore the influence of the impedance C_0_. The resistance R_1_ generally has a few MΩ, the capacitance C_1_ hundreds of nF, ω/ 2ᴫ a few kHz (ω≈ω_nx_), so R_1_≫1/ωC_1_, the output voltage P(t) is described by:

(17) $$P(t)=I_{1}(t)R=I(t)R\frac{R_{1}}{R_{1}+1/j\omega C_{1}}\approx V_{s}RK_{xc}\dot{x}$$

**Figure 3.**
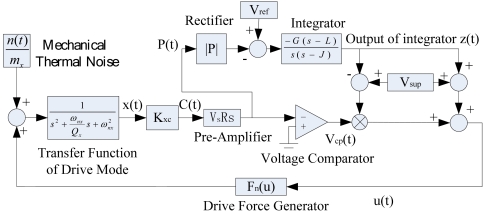
The frame of the closed-loop driving.

Figure 3 is the frame of the closed-loop driving. In this figure s is a complex variable. V_s_, V_ref_ and V_sup_ are the direct current biases. L and J are the zero and the pole of the integrator separately. G is the gain of the integrator. In order to improve the precision and the stability of the closed-loop driving, the Q-factor of the drive-mode should be increased (the SMG is executed in vacuum encapsulation), while the well closed-loop control should be achieved. The closed-loop control must meet such two conditions: 1. The phase of the whole loop θ=2nπ (n is an integer); 2. The gain of the whole loop A>1.

A new closed-loop drive scheme which decouples the phase and the gain of the closed-loop driving system is adopted in the SMG, so that the phase and the gain can be optimized, respectively. The gain of the whole closed-loop system is controlled by the branch circuit above, and the phase is controlled by the branch circuit below. These two branch circuits respectively fulfill the two conditions of closed-loop control, adjusting and optimizing the closed-loop parameter separately. The “voltage comparator” is the key component of the closed-loop driving. The output of the “voltage comparator”, with an invariable output amplitude, only reserves the phase information of the input signal, so the phase conditions of the closed-loop are isolated from the gain conditions. Except the drive mode of SMG and “voltage comparator”, with the suppose that the phase of the other parts is fixed, when the phase of the “voltage comparator” is changed, the vibrating frequency of the closed-loop system will depart from the resonant frequency of the proof mass, so the phase condition controls the frequency of the driving displacement. It is obvious that the gain branch controls the amplitude of the driving displacement. According to Equations (34) and (35) (See Section 5), ignoring the influence of the random processes n_1_(t) and n_2_(t), we can know that average amplitude *ā* and average phase *φ̄* are decoupled, so the phase branch and the gain branch can be optimized respectively and the new closed-loop drive scheme is succeeded.

To make sure that the harmonic frequency of the closed-loop system equals, or gets close to the resonant frequency of the proof mass, that is to say, the phase of the whole loop θ = 2nπ(n is an integer). When ω_d_=ω_nx_, the phase-shift of the drive displacement x(t) comparing to the drive force F_e_ is -π/2(See Equation (5)), the phase-shift of the output voltage of pre-amplifier P(t) comparing to drive displacement x(t) is -π/2 and the phase-shift of the output voltage of the “voltage comparator” V_cp_(t) comparing to output voltage of preamplifier P(t) is −π. The other parts, which in fact all have tiny phase errors, have no phase-shifts, so the closed-loop control meets the phase conditions. In this way, it is hoped that the above closed-loop phase errors should be as tiny as possible. Various techniques are generally utilized in an effort to reduce closed-loop phase error, or drift, in servo circuits, such as amplifier circuits utilizing an operational amplifier. One such technique includes the addition of one or more zeros (i.e., a lead filter) in cascade with the open-loop gain of the operational amplifier in order to flatten the open-loop gain over a portion of the frequency band, generally resulting in only moderate closed-loop error reduction and also compromising stability. Another technique for reducing gain and phase errors is to increase the gain-bandwidth product associated with the operational amplifier. However, use of this technique is limited by the gain-bandwidth product of commercially available operational amplifiers as well as by the acceptable increased power dissipation associated with higher performance operational amplifiers. However, a Phase-Corrected Amplifier Circuit can be used to remove the closed-loop phase error [13]. An amplifier circuit having a bandpass circuit in cascade with the forward loop gain is provided, with the bandpass circuit having a transfer function approximating one plus a bandpass characteristic, the passband of which corresponds to the information band. This arrangement increases the open-loop gain of the amplifier circuit around the information frequency without affecting the open-loop gain at DC and crossover so as to reduce phase and gain errors around the information frequency.

In Figure 1, the drive capacitance and the electrical potential energy stored in the capacitance is described as:

(18) $$C_{d1}(t)={ɛ}_{0}\frac{h(x_{0}+x(t))}{d}$$

(19) $$U=\frac{1}{2}C_{d1}(t)V^{2}$$

where ε_0_ is the permittivity, h the thickness of the comb fingers, x_0_ the overlap length of the fingers, and d the width of the gap between fingers. According to Figure 3, the electrostatic drive force is described by:

(20) $$\begin{array}{ll} F_{e} & =\frac{1}{2}\frac{\partial C_{d1}(t)}{\partial x}V^{2}=\frac{1}{2}\frac{\partial C_{d1}(t)}{\partial x}[(V_{\sup}-z(t))V_{cp}(t)+(V_{\sup}+z(t))]\\ & =\frac{1}{2}\frac{\partial C_{d1}(t)}{\partial x}{\left(V_{\sup}+z(t)\right)}^{2}+\frac{\partial C_{d1}}{\partial x}(V_{\sup}^{2}-z{\left(t\right)}^{2})V_{cp}(t)+\frac{1}{2}\frac{\partial C_{d1}(t)}{\partial x}{\left(V_{\sup}-z(t)\right)}^{2}V_{cp}{\left(t\right)}^{2} \end{array}$$

where $\frac{\partial C_{d1}}{\partial x}$ is the differential of driving capacitance to displacement, the z(t) is a DC and V_cp_(t) is a AC with the frequency near the resonant frequency ω_nx_. The first term of Equation (20) is a constant and does not contribute to the oscillating driving force. Since the amplitude of the AC driving voltage is chosen to be much smaller than the bias voltage, the third term of Equation (20) is much smaller than the second term and can be neglected. Therefore, under proper bias, the driving force is approximately proportional to the AC driving voltage.

According to Figure 3, the drive-mode of the SMG is modeled as a second-order spring-mass-damper system with a dynamic behavior described by:

(21) $$\ddot{x}+\frac{\omega_{nx}}{Q_{x}}\dot{x}+\omega_{nx}^{2}x=F_{n}(u)+\frac{n}{m_{x}}$$

where F_n_(u) is electrostatic drive force $\left(F_{n}(u)=F_{e}/m_{x}\approx \frac{\partial C_{d1}}{\partial x}(V_{\sup}^{2}-z{\left(t\right)}^{2})V_{cp}(t)/m_{x}\right)$, and suppose z(t)≥0.

Simplifying the integrator (See Figure 3) into the basic integral function, so:

$$-G\int_{0}^{t}\left(V_{\text{ref}}-|P(t)|\right)dt=z(t)$$

i.e.:

(22) $$\dot{z}(t)=-G(V_{\text{ref}}-V_{s}R|K_{xc}||\dot{x}|)$$

where |*ẋ*| is modulus of the driving velocity and:

$$P(t)=V_{s}RK_{xc}\dot{x}$$

So the output of the “voltage comparator” is:

(23) $$V_{cp}(t)=-\frac{P(t)}{\left|P(t)\right|}=-\frac{K_{xc}\dot{x}}{\left|K_{xc}||\dot{x}\right|}$$

In summary, the entire closed-loop driving system, shown in Figure 3, are described by Equations (24)∼(25):

(24) $$\ddot{x}+R_{eq}\dot{x}+\omega_{nx}^{2}x=\frac{n}{m_{x}}$$

(25) $$\dot{z}(t)=-G(V_{\text{ref}}-V_{s}R|K_{xc}||\dot{x}|)$$

where R_eq_ $\left(R_{eq}=\frac{\omega_{nx}}{Q_{x}}+(V_{\sup}^{2}-z{\left(t\right)}^{2})\frac{\partial C_{d1}}{\partial x}\frac{K_{xc}}{|K_{xc}||\dot{x}|m_{x}}\right)$ is equivalent damper. Equations (24) and (25) show the closed-loop system model. With the ignorance of the influence of the random process n(t), Equation (24) shows a free spring-mass-damper oscillating system and Equation (25) shows the control principle of the integrator. The closed-loop system reduces the system damper R_eq_ through adjusting the output of the integrator z(t). When the equivalent damper R_eq_ is bigger than the zero(e.g.R_eq_>0), the gain control loop reduces the output of the integrator z(t) and then the equivalent damper R_eq_ will decrease near the zero (R_eq_≈0, $\frac{\partial C_{d1}}{\partial x}$ is inverse-phase with K_xc_). When the equivalent damper R_eq_ is smaller than the zero (e.g.R_eq_<0), the gain control loop enhances the output of the integrator z(t) and thus, the equivalent damper will increase near the zero (R_eq_≈0). So the closed-loop system shows approximately an undamped-free vibration with the invariable amplitude and frequency(e.g. the resonant frequency ω_nx_). However, the random process n(t) will influence the stability of the driving amplitude and frequency.

## 5. Stochastic Averaging of the Gyroscope Dynamics

After the vacuum encapsulation, the quality factor of the drive-mode Q_x_ becomes very big. The drive-mode of the SMG can be equivalent to a band-pass filter. Only the displacement with the resonant frequency ω_nx_ can be magnified and the other harmonics are attenuated greatly. So the transient displacement can be simplified into a pure sin component. In order to analyze the transient behavior of the system, the driving displacement of the SMG is defined as [14]:

(26) $$x=a(t)\cos(\omega_{nx}t+\phi (t))$$

where *a*(*t*) is the amplitude, and *φ*(*t*) is the phase of the driving displacement signal respectively.

Differentiating Equation (26) with respect to time gives the velocity:

$$\dot{x}=-a\omega_{nx}\sin(\omega_{nx}t+\phi )+\dot{a}\cos(\omega_{nx}t+\phi )-a\dot{\phi }\sin(\omega_{nx}t+\phi )$$

One of the equations used to determine *a* and *φ* is obtained by assuming that the sum of the last two terms can be set equal to zero:

(27) $$\dot{a}\cos(\omega_{nx}t+\phi )-a\dot{\phi }\sin(\omega_{nx}t+\phi )\equiv 0$$

Thus, the velocity equation becomes:

(28) $$\dot{x}=-a\omega_{nx}\sin(\omega_{nx}t+\phi )$$

The acceleration is obtained by differentiating Equation (28) with respect to time:

(29) $$\ddot{x}=-\dot{a}\omega_{nx}\sin(\omega_{nx}t+\phi )-a\omega_{nx}(\omega_{nx}+\dot{\phi })\cos(\omega_{nx}t+\phi )$$

Substituting Equations (26), (28) and (29) into Equation (24) yields:

(30) $$-(\dot{a}\omega_{nx}+a\frac{\omega_{nx}^{2}}{Q_{x}})\sin(\omega_{nx}t+\phi )-a\omega_{nx}\dot{\phi }\cos(\omega_{nx}t+\phi )=\frac{\partial C_{d1}}{\partial x}(V_{\sup}+z(t))(V_{\sup}-z(t))\frac{K_{xc}a\sin(\omega_{nx}t+\phi )}{|K_{xc}||a\sin(\omega_{nx}t+\phi )|m_{x}}+\frac{n}{m_{x}}$$

Substituting Equation (28) into Equation (25), and suppose *a*≠0, according to Equation (27) and Equation (30), separate *ȧ*, *φ̇* yields:

(31) $$\dot{a}=-a\frac{\omega_{nx}}{Q_{x}}\sin^{2}(\omega_{nx}t+\phi )-\frac{\partial C_{d1}}{\partial x}\frac{K_{xc}a\sin^{2}(\omega_{nx}t+\phi )(V_{\sup}+z)(V_{\sup}-z)}{|K_{xc}||a\sin(\omega_{nx}t+\phi )|m_{x}\omega_{nx}}-\frac{n\sin(\omega_{nx}t+\phi )}{m_{x}\omega_{nx}}$$

(32) $$\dot{\phi }=-\frac{\omega_{nx}}{Q_{x}}\sin(\omega_{nx}t+\phi )\cos(\omega_{nx}t+\phi )-\frac{\partial C_{d1}}{\partial x}\frac{K_{xc}\sin(\omega_{nx}t+\phi )\cos(\omega_{nx}t+\phi )(V_{\sup}+z)(V_{\sup}-z)}{|K_{xc}||a\sin(\omega_{nx}t+\phi )|m_{x}\omega_{nx}}-\frac{n}{am_{x}\omega_{nx}}\cos(\omega_{nx}t+\phi )$$

(33) $$\dot{z}(t)=-G(V_{\text{ref}}-V_{s}R|K_{xc}||a\omega_{nx}\sin(\omega_{nx}t+\phi )|)$$

It should be noted that Equations (31)∼(33) are the exact differential equations describing the evolution of the amplitude and phase of the driving displacement, as well as that of the output states of the integrator. However, these equations are difficult to analyze because they are substantially non-autonomous. It is evident that instantaneous phase ω_nx_t evolves much faster than the other variables, such as *a, φ, z* and functions sin(ω_nx_t+*φ*) and cos(ω_nx_t+*φ*) are almost periodic. Within a period of these sinusoidal functions, variables other than ω_nx_t change very little. Hence, it is possible to apply the averaging method to the non-autonomous system described by Equations (31)∼(33) and approximate it by an autonomous system [14-15].

As pointed out above, instantaneous phase ω_nx_t is regarded as an independent variable and the differential equations Equations (31)∼(33) are averaged, with respect to ω_nx_t, over the interval [-π, π]. The averaged autonomous equations are:

(34) $$\dot{\bar{a}}=-\frac{\bar{a}\omega_{nx}}{2Q_{x}}-\frac{\partial C_{d1}}{\partial x}\frac{2K_{xc}(V_{\sup}+\bar{z})(V_{\sup}-\bar{z})}{\pi |K_{xc}|m_{x}\omega_{nx}}+\frac{n_{1}}{\sqrt{2}m_{x}\omega_{nx}}$$

(35) $$\dot{\bar{\phi }}=\frac{n_{2}}{\sqrt{2}\bar{a}m_{x}\omega_{nx}}$$

(36) $$\dot{\bar{z}}=-G(V_{\text{ref}}-\frac{2V_{s}R|K_{xc}|\omega_{nx}\bar{a}}{\pi })$$

where the bars denote averaged variables. The random processes n_1_(t) and n_2_(t) are independent white noise with the same intensity as n (t) [10]. Equations (34)∼(36) describe approximately how the displacement amplitude and phase evolve with time. According to Equations (34) and (35), it is evident that average amplitude *ā* and average phase *φ̄* are decoupled – when one of them is changed, the other does not change, so the phase branch and the gain branch can be optimized respectively.

Ignoring the influence of the random processes n_1_(t) and n_2_(t), the equilibrium of the averaged system described by Equations (34)∼(36) is:

(37) $${\bar{a}}_{o}=\frac{\pi V_{\text{ref}}}{2V_{s}R|K_{xc}|\omega_{nx}}$$

(38) $${\bar{z}}_{o}=\sqrt{V_{\sup}^{2}+\frac{\pi^{2}V_{\text{ref}}m_{x}\omega_{nx}}{8Q_{x}V_{s}RK_{xc}\frac{\partial C_{d1}}{\partial x}}}$$

When the power is switched on, the system stabilizes finally in equilibrium. According to Equation (37), it is evident that the equilibrium of the average amplitude *ā_o_* is independent from the quality factor Q_x_, so the change of quality factor resulting from the variety of temperature and pressure does not impact on the equilibrium of the average amplitude *ā_o_*, which is very important in the practical application. The speciality of this system is obviously different from the literature [16].

Ignoring the influence of the random processes n_1_(t) and n_2_(t), the Jacobian matrix of the nonlinear dynamic system of Equations (34)∼(36) at equilibrium is:

(39) $${\frac{\partial f}{\partial (\bar{a},\bar{z})}}_{\left({\bar{a}}_{o},{\bar{z}}_{o}\right)}=\left[\begin{array}{cc} -\frac{\omega_{nx}}{2Q_{x}} & \frac{4K_{xc}}{\pi |K_{xc}|m_{x}\omega_{nx}}\frac{\partial C_{d1}}{\partial x}\sqrt{V_{\sup}^{2}+\frac{\pi^{2}V_{\text{ref}}m_{x}\omega_{nx}}{8Q_{x}V_{s}RK_{xc}\frac{\partial C_{d1}}{\partial x}}}\\ \frac{2GV_{s}R|K_{xc}|\omega_{nx}}{\pi } & 0 \end{array}\right]$$

and its characteristic equation is:

(40) $$\gamma^{2}+\frac{\omega_{nx}}{2Q_{x}}\gamma -\frac{\partial C_{d1}}{\partial x}\frac{8GV_{s}RK_{xc}}{\pi^{2}m_{x}}\sqrt{V_{\sup}^{2}+\frac{\pi^{2}V_{\text{ref}}m_{x}\omega_{nx}}{8Q_{x}V_{s}RK_{xc}\frac{\partial C_{d1}}{\partial x}}}=0$$

where *γ* is the variable of the characteristic equation. All the eigenvalues of the linearized averaged system are asymptotically stable, if and only if all coefficients is positive. It is evident Equation (40) satisfies this condition because $\frac{\partial C_{d1}}{\partial x}$ is inverse-phase with K_xc_. In order to make sure that the square root is in existence, the inequation hereinafter must be satisfied:

$$V_{\sup}^{2}+\pi^{2}V_{\text{ref}}m_{x}\omega_{nx}/(8Q_{x}V_{s}RK_{xc}\frac{\partial C_{d1}}{\partial x})\geq 0$$

i.e.:

(41) $$V_{\text{ref}}\leq \frac{-8V_{\sup}^{2}Q_{x}V_{s}RK_{xc}}{\pi^{2}m_{x}\omega_{nx}}\frac{\partial C_{d1}}{\partial x}=V_{\text{refo}}$$

where the V_ref_ is the referring DC voltage used as a reference to the amplitude of the pre-amplifier output voltage. The V_refo_ is the criterion voltage. When V_ref_ < V_refo_, the gain control branch works normally. When V_ref_ >V_refo_, the gain control branch loses the control ability. Because the Equations (34)∼(36) are extremely complex, the averaged system is linearized in the equilibrium, i.e.:

(42) $${\dot{\bar{a}}}_{n}=-\frac{\omega_{nx}}{2Q_{x}}{\bar{a}}_{n}+\frac{n_{1}}{\sqrt{2}m_{x}\omega_{nx}}+\frac{4K_{xc}}{\pi |K_{xc}|m_{x}\omega_{nx}}\frac{\partial C_{d1}}{\partial x}\sqrt{V_{\sup}^{2}+\frac{\pi^{2}V_{\text{ref}}m_{x}\omega_{nx}}{8Q_{x}V_{s}RK_{xc}\frac{\partial C_{d1}}{\partial x}}}{\bar{z}}_{n}$$

(43) $$\dot{\bar{\phi }}=\frac{n_{2}}{\sqrt{2}\bar{a}m_{x}\omega_{nx}}$$

(44) $${\dot{\bar{z}}}_{n}=\frac{2GV_{s}R|K_{xc}|\omega_{nx}}{\pi }{\bar{a}}_{n}$$

where *ā_n_* = *ā* − *ā_o_, z̄_n_* = *z̄* − *z̄_o_*. *ā_n_* can be expressed in Laplace transform domain as:

(45) $${\bar{A}}_{n}(s)=\frac{\frac{N_{1}(s)}{\sqrt{2}m_{x}\omega_{nx}}s}{s^{2}+\frac{\omega_{nx}}{2Q_{x}}s+k_{n}}$$

where $k_{n}=-\frac{8GV_{s}RK_{xc}\frac{\partial C_{d1}}{\partial x}}{\pi^{2}m_{x}}\sqrt{V_{\sup}^{2}+\frac{\pi^{2}V_{\text{ref}}m_{x}\omega_{nx}}{8Q_{x}V_{s}RK_{xc}\frac{\partial C_{d1}}{\partial x}}}$, *Ā_n_*(*s*) is Laplace transform of *ā_n_*, and *N*_1_(*s*) is Laplace transform of the *n*_1_, so the steady-state spectral density for the noise component of the *ā_n_* is:

(46) $$S_{{\bar{a}}_{n}}(\omega )=\frac{\omega^{2}}{2m_{x}^{2}\omega_{nx}^{2}\left({\left(k_{n}-\omega^{2}\right)}^{2}+\frac{\omega_{nx}^{2}\omega^{2}}{4Q_{x}^{2}}\right)}S_{n_{1}}(\omega )$$

So the noise power spectrum is:

$$<{\bar{a}}_{n}^{2}>=\frac{1}{2\pi }\int_{-\infty }^{+\infty }S_{{\bar{a}}_{n}}(\omega )d\omega =\frac{k_{B}T}{k_{x}}$$

The RMS noise amplitude due to thermal noise is:

(47) $${\bar{a}}_{n}=\sqrt{<{\bar{a}}_{n}^{2}>}=\sqrt{\frac{k_{B}T}{k_{x}}}=\sqrt{\frac{k_{B}T}{m_{x}{\omega^{2}}_{nx}}}$$

Comparing Equation (14) with Equation (47), we know the RMS noise amplitude due to thermal noise in the opened-loop driving is equal to that in the closed-loop driving, so the closed-loop driving does not reduce the RMS noise amplitude due to thermal noise. By the way, the RMS noise amplitude is independent from the quality factor Q_x_.

According to *ā* = *ā_o_* + *ā_n_* ≈ *ā_o_*, Equation (43) can be rewritten as:

(48) $${\bar{\omega }}_{n}=\dot{\bar{\phi }}=\frac{n_{2}}{\sqrt{2}{\bar{a}}_{o}m_{x}\omega_{nx}}$$

where *ω̄_n_* is the noise frequency, so the power spectral density of the noise frequency *ω̄_n_* is

(49) $$S_{{\bar{\omega }}_{n}}(\omega )=\frac{1}{2{\bar{a}}_{o}^{2}m_{x}^{2}\omega_{nx}^{2}}S_{n_{2}}(\omega )$$

Suppose the work bandwidth is f_B_Hz, the noise power is:

(50) $$<{\bar{\omega }}_{n}^{2}>=\frac{1}{2\pi }\int_{-2\pi f_{B}}^{+2\pi f_{B}}S_{{\bar{\omega }}_{n}}(\omega )d\omega =\frac{2f_{B}k_{B}T\omega_{nx}}{{\bar{a}}_{o}^{2}k_{x}Q_{x}}$$

The RMS noise frequency due to thermal noise is:

(51) $${\bar{\omega }}_{n}=\sqrt{<{\bar{\omega }}_{n}^{2}>}=\frac{\sqrt{2}}{{\bar{a}}_{o}}\sqrt{\frac{f_{B}k_{B}T\omega_{nx}}{k_{x}Q_{x}}}=\frac{\sqrt{2}}{{\bar{a}}_{o}}\sqrt{\frac{f_{B}k_{B}T}{\omega_{nx}m_{x}Q_{x}}}$$

It is useful to reduce RMS noise frequency by increasing quality factor Q_x_ and drive amplitude *ā_0_*.

## 6. Experiment and Simulation

### 6.1. Simulation

**Figure 4.**
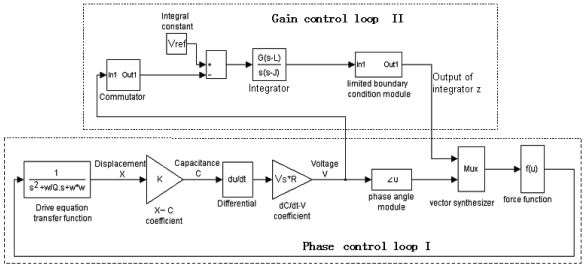
The Matlab simulation frame of the closed-loop driving.

In order to validate the feasibility of closed-loop driving and confirm the validity of the averaged equation and its stable criterion, the closed-loop driving in Figure 3 is simulated according to parameters shown in Table 1. Figure 4 is the Matlab simulation frame of the closed-loop driving.

**Figure 5.**
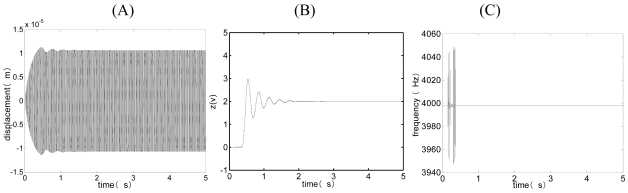
The simulation curves of closed-loop driving when *V_ref_* = 0.5 < *V_refo_*. (A) The curve of the drive displacement with time. (B) The curve of the output of integrator z(t) with time. (C) The curve of the vibrating frequency of the drive-mode with time.

**Figure 6.**
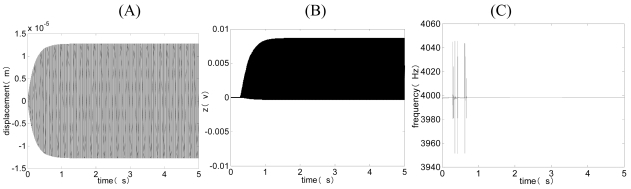
The simulation curves of closed-loop driving when *V_ref_* = 0.7 > *V_refo_*. (A) The curve of the drive displacement with time. (B) The curve of the output of integrator z(t) with time. (C) The curve of the vibrating frequency of the drive-mode with time.

Figures 5 and 6 are the simulation curves for closed-loop driving when *V_ref_* = 0.5 < *V_refo_* and *V_ref_* = 0.7 > *V_refo_* [see Equation (41)]. It can be concluded from Figures 5(C) and 6(C) that the system resonant frequency approximately equals to the natural frequency of drive-mode after the system is stabilized, which indicates that the whole loop's phase θ = 2nл(n is an integer)and the phase branch circuit below is controlled accurately. When *V_ref_* < *V_refo_*, according to Figure 5 and Equation (37), the driving displacement goes along with the increase of *V_ref_* and the gain branch circuit above is controlled accurately. When *V_ref_* = *V_refo_*, the driving displacement reaches the maximum. When *V_ref_* increases persistently, that is to say, *V_ref_* > *V_refo_*, according to Figure 6, the output of the integrator z≈0 and the driving displacement does not go along with the increase of *V_ref_* and the gain branch circuit above does not work normally. The curve of the transient course of drive displacement along with the change of time goes smooth and the overshoot disappeares. Comparing Figure 5(A) to Figure 6(A), when *V_ref_* < *V_refo_*, we can see that the bigger the *V_ref_* is, the shorter the transient time is. When *V_ref_* > *V_refo_*, the above gain branch circuit does not work normally and the closed-loop system loses the adjustable ability, with the overshoot fading away.

**Figure 7.**
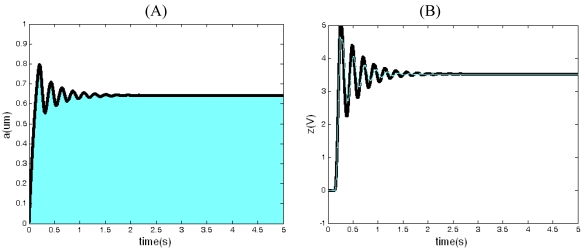
Closed-loop response (gray) and averaged equation simulation (black) when *V_ref_* = 0.3 < *V_refo_*. (A) The curve of the drive displacement with time. (B) The curve of the output of integrator z(t) with time.

**Figure 8.**
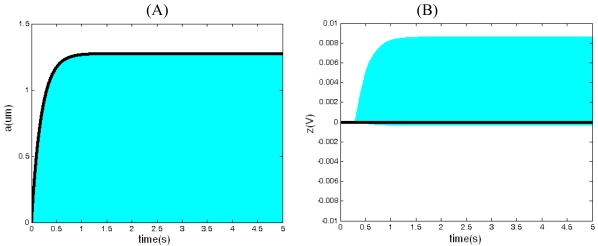
Closed-loop response (gray) and averaged equation simulation (black) when *V_ref_* = 0.7 > *V_refo_*. (A) The curve of the drive displacement with time. (B) The curve of the output of integrator z(t) with time.

Figures 7 and 8 are the simulated closed-loop response and averaged equation simulation curves when *V_ref_* = 0.3 < *V_refo_* and *V_ref_* = 0.7 > *V_refo_*. The simulation result indicates the envelope curve of the closed-loop response is basically the same as the curve of the averaged equation. The tiny differences come from the simplification of the integrator and truncation error in simulation. From Figure 8, when *V_ref_* = 0.7 > *V_refo_*, we can see that the envelope curve of the closed-loop response is also basically the same as the curve of averaged equation. Meanwhile, z ≈0, revealing that the above gain branch circuit does not work normally, which is in agreement with the practical situation. Thus, the averaged equation can be applied to the situations when *V_ref_* > *V_refo_* through the limit of the working scale of z(t).

**Figure 9.**
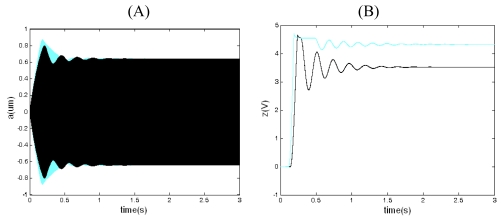
The simulation curve of closed-loop driving in Q_x_=2,500 (black) and Q_x_=5,000 (gray) when *V_ref_* = 0.3 < *V_refo_*. (A) The curve of the drive displacement with time. (B) The curve of the output of integrator z(t) with time.

**Figure 10.**
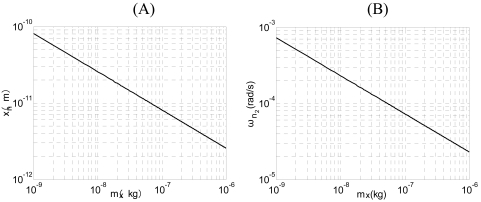
The influence of thermal noise to driving performance when ω_nx_= 25,120 (rad/s), T=300 K, f_B_=100 Hz, Q_x_=2,500, *ā_o_* = 5 μm. (A) The RMS noise displacements with driving mass. (B) The RMS noise frequency with driving mass.

In Figure 9, the simulation curves of closed-loop driving between Q_x_=2,500 and Q_x_=5,000 are compared. From Figure 9(A), we can see that although the quality factor increases, the amplitude of the driving displacement, which is in agreement with the conclusion derived from Equation (37), doesn't change. That is to say, the displacement amplitude has nothing to do with the quality factor. From Figure 9(B), we can see that the augment of quality factor results in the growth of output of the integrator z(t), which is accordant with the Equation (38). According to Equation (47) and Equation (51), when ω_nx_= 25,120 (rad/s), T=300 K, f_B_=100 Hz, Q_x_=2,500, *ā_o_* = 5 μm, the influence of thermal noise on driving performance in different drive proof masses is shown in Figure 10. From Figure 10, we can see that the RMS noise displacement and the RMS noise frequency decrease with the increase of drive proof mass.

### 6.2. Experiment

**Figure 11.**
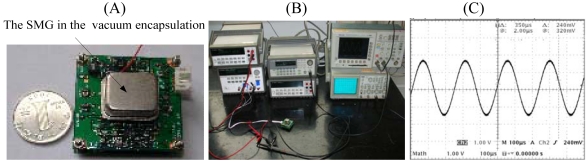
The experiment of closed-loop driving. (A) The PCB circuitry of SMG. (B) The test setup environment. (C)The vibrating waveshape of closed loop driving signal.

**Figure 12.**
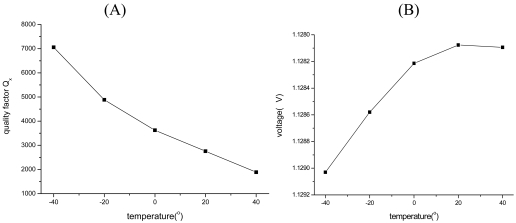
The experiment results of amplitude control with temperature. (A) The curve of the quality factor of the drive-mode Q_x_ with temperature. (B) The signal amplitude of closed loop driving with temperature.

The whole experiment circuit is constructed on the idea of Figure 3 and shown in Figure 11. The experiment results show that the SMG achieves closed-loop driving and the drive frequency works around the natural frequency of SMG all the time. The vibrating waveshape of the closed loop driving signal is shown in Figure 11(C). It is practical to find a better working point for SMG by adjusting z(t) in Figure 3. The experiment results of amplitude control with temperature are shown in Figure 12. According to the Equation (5), when ω_d_=ω_nx_, the amplitude of driving displacement is proportional with the quality factor Q_x_, so the change of quality factor, due to the variety of temperature and pressure, impacts directly on the amplitude of driving displacement in the open-loop driving. But the new closed loop driving is immunized to the change of the quality factor [see Equation (37) and Equation (47)]. With a temperature increase from -40°C to +40°C, the quality factor of the drive-mode Q_x_ decreases about 2.6 times [see Figure 12(A)], while the drive amplitude signal increases by only 0.083% in the closed loop driving [see Figure 12(B)].

**Figure 13.**
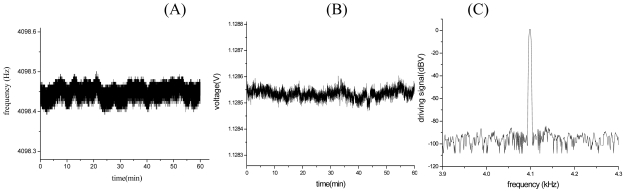
The experiment results of closed-loop driving.(A) Frequency drift of driving signal for 1 h. (B) Amplitude drift of driving signal for 1h. (C) Frequency spectrum of driving signal.

The experiment results of closed-loop driving are shown in Figure 13. Figure 13(A) and Figure 13(B) are the frequency drift and amplitude drift of driving signal for 1 h respectively. The experiment results show that the standard deviation of drive frequency is 0.0205 Hz, with relative drift 5.0 ppm, the standard deviation of the amplitude 0.0165 mV and relative drift 14.7 ppm, respectively. Figure 13(C) is the frequency spectrum of driving signal. The driving signal is about 100 dB bigger than the ground noise, which is accordance with the result of the Figure 13(B). As a result, both stabilities of driving frequency and amplitude are rather high and the closed loop control is very successful. According to Figure 10, when m_x_=2.89×10^-7^(kg), *ā_o_* = 5um, ω_nx_= 25,120 (rad/s), we can find that the relative noise of drive frequency is approximately 0.0119 ppm and the relative noise of the amplitude is approximately 0.8 ppm, respectively. The noise comparison is shown in Table 2. So, in this situation, the electrical noise of closed-loop driving circuitry is bigger than the mechanical-thermal noise. With the driving mass decreasing, the mechanical-thermal noise may get bigger than the electrical noise of closed-loop driving circuitry (see Figure 10).

## 7. Discussion and Conclusions

The mechanical thermal noise on drive-mode is discussed, and then stochastic averaging is used to develop a model for the “slow” dynamics that represent the driving amplitude and frequency of the SMG. Both the steady-state and transient response of the model are obtained by stochastic averaging. The spectral density of the random error due to thermal noise on drive-mode is also derived. By calculating and comparing the RMS noise amplitude due to thermal noise both in the opened-loop driving and in the closed-loop driving, we find that the closed-loop driving does not reduce the RMS noise amplitude. We observe that the RMS noise frequency can be reduced by increasing the quality factor and drive amplitude in the closed-loop driving system. The experiment and simulation validate the feasibility of closed-loop driving and confirm the validity of the averaged equation. The experiment and simulation results indicate the electrical noise of closed-loop driving circuitry is bigger than the mechanical-thermal noise and with the driving mass decreasing, the mechanical-thermal noise may get bigger than the electrical noise of closed-loop driving circuitry.

## Figures and Tables

**Table 1.** The value of simulation parameter.

**Parameter**	**value(unit)**
Qx	2,500
G	999
Kxc	2.92×10^-8^ (F/m)
Vs	10 (V)
R	10 (MΩ)
Vsup	5 (V)
Vrefo	0.596 (V)
L	-50
J	-55
|∂C/∂x|	1.46×10^-6^ (F/m)
ωnx	25120 (rad/s)
mx	2.89×10^-7^ (kg)

**Table 2.** The noise comparison.

**Simulation thermal noise**			**Measure noise(Driving signal)**		
mx = 2.89×10^-7^(kg), *ā_o_* = 5μm, ωnx = 25,120 (rad/s)					
	Absolute value	Relative value		Absolute value	Relative value
Thermal noise frequency	0.0000476 Hz	0.0119 ppm	Noise frequency	0.0205 Hz	5.0 ppm
Thermal noise amplitude	4×10^-12^ m	0.8 ppm	Noise amplitude	0.0165 mV	14.7 ppm
